# Dietary Polyphenols: Review on Chemistry/Sources, Bioavailability/Metabolism, Antioxidant Effects, and Their Role in Disease Management

**DOI:** 10.3390/antiox13040429

**Published:** 2024-03-30

**Authors:** Mithun Rudrapal, Gourav Rakshit, Ravi Pratap Singh, Samiksha Garse, Johra Khan, Soumi Chakraborty

**Affiliations:** 1 Department of Pharmaceutical Sciences, School of Biotechnology and Pharmaceutical Sciences, Vignan’s Foundation for Science, Technology & Research (Deemed to be University), Guntur 522213, India; 2 Department of Pharmaceutical Sciences & Technology, Birla Institute of Technology, Ranchi 835215, India; phdph10052.20@bitmesra.ac.in (G.R.); phdph10007.19@bitmesra.ac.in (R.P.S.); mph10043.22@bitmesra.ac.in (S.C.); 3 School of Biotechnology and Bioinformatics, D Y Patil Deemed to be University, Navi Mumbai 400614, India; samiksha.garse@dypatil.edu; 4 Department of Medical Laboratory Sciences, College of Applied Medical Sciences, Majmaah University, Al Majmaah 11952, Saudi Arabia; j.khan@mu.edu.sa

**Keywords:** dietary polyphenols, health benefits, antioxidant effects, bioavailability and metabolism, disease management

## Abstract

Polyphenols, as secondary metabolites ubiquitous in plant sources, have emerged as pivotal bioactive compounds with far-reaching implications for human health. Plant polyphenols exhibit direct or indirect associations with biomolecules capable of modulating diverse physiological pathways. Due to their inherent abundance and structural diversity, polyphenols have garnered substantial attention from both the scientific and clinical communities. The review begins by providing an in-depth analysis of the chemical intricacies of polyphenols, shedding light on their structural diversity and the implications of such diversity on their biological activities. Subsequently, an exploration of the dietary origins of polyphenols elucidates the natural plant-based sources that contribute to their global availability. The discussion extends to the bioavailability and metabolism of polyphenols within the human body, unraveling the complex journey from ingestion to systemic effects. A central focus of the review is dedicated to unravelling the antioxidant effects of polyphenols, highlighting their role in combating oxidative stress and associated health conditions. The comprehensive analysis encompasses their impact on diverse health concerns such as hypertension, allergies, aging, and chronic diseases like heart stroke and diabetes. Insights into the global beneficial effects of polyphenols further underscore their potential as preventive and therapeutic agents. This review article critically examines the multifaceted aspects of dietary polyphenols, encompassing their chemistry, dietary origins, bioavailability/metabolism dynamics, and profound antioxidant effects. The synthesis of information presented herein aims to provide a valuable resource for researchers, clinicians, and health enthusiasts, fostering a deeper understanding of the intricate relationship between polyphenols and human health.

## 1. Introduction

The overall lifestyle of an individual is compromised due to professional competition, irregular food intake, and social-behavioral habits like drinking and smoking [1]. Their significant impact on the biological processes has led to disorders like hypertension, diabetics, aging, neurodegenerative diseases, heart diseases, etc. [1,2]. Such irregularities trigger the production of free radicals causing oxidative stress, a lead mechanism resulting in the imbalance between prooxidants and antioxidants [2]. Many clinicians and scientists identified polyphenols to be the key component with anti-oxidant effects and play a major role in overcoming these disorders [1]. Being the secondary metabolite of plants, they are abundant in nature with a wide range of structural differences, which has led to their categorization [3].

Antioxidants attenuate the effects of the activities of pro-oxidants or free radicals by quenching their oxidative activity. These free radicals are species capable of non-dependent existence with one or more unpaired electrons [4]. Oxidative stress is characterized by the generation of reactive oxygen species [5] that may be involved in cellular reactions with DNA, lipids, and proteins, among other macromolecules [6]. Such cellular reactions have been attributed to the cause of most diseases, such as inflammation, cancer, brain dysfunction, cardiovascular disease, organ failures, and the general decline in the human immune system [7]. Even though various synthetic antioxidant agents have been available over the years, their cytotoxicity has numerous side effects [8]. Due to this, there is a need for various kinds of phyto-antioxidants that are fruit, vegetable, and herbal plant-based, have hardly any side effects, and are cheaper than synthetic agents to be used as a source of alternative medicine [9]. These phyto-antioxidants effectively inhibit oxidative species involved in various diseases, offering potential therapeutic benefits against conditions like cardiovascular disease, neurodegenerative disorders, and cancer. Additionally, their anti-inflammatory properties make them promising candidates for managing inflammatory conditions such as obesity and type 2 diabetes mellitus [8].

Polyphenols stand out as the leading stars in the realm of pharmacological therapeutics, showcasing their prowess as primary components in our daily dietary intake [10]. Aside from being an essential antioxidant agent, this group of compounds has been widely reported to possess anti-allergic, antihypertensive, anti-inflammatory, anticancer, antiviral, and antimicrobial potencies [11] of which most of these biological activities are consequences of the antioxidative effect of this class of organic molecules. As a group of compounds that exhibit antioxidant activities, polyphenols prevent aging [12] and degenerative diseases such as cardiovascular, cancer and neurodegenerative diseases [13]. Most fruits and medicinal plants with high polyphenolic content exhibit antioxidant potency. Even though the basic structural unit in polyphenols is the phenolic ring [14], they are composed of a wide range of complex structures with structural features that enhance their antioxidant potency [15].

We posit that the heterogeneity in molecular architecture inherent to polyphenols underpins their classification and modulates their source-dependent bioavailability, thereby dictating their physiological and pharmacokinetic profiles. This structural variation is hypothesized to be pivotal in mediating the interaction of polyphenols with cellular pathways, which is crucial for their metabolic assimilation and therapeutic efficacy in mitigating various pathologies. Furthermore, the distinct chemical signatures of polyphenols are anticipated to confer differential impacts on food quality and commercial viability, as well as on the epigenome, suggesting a potential for modulating gene expression. Consequently, this hypothesis integrates the chemical individuality of polyphenols with their biotransformation, health-promoting attributes, economic significance, and epigenetic influence, proposing a comprehensive and synergistic impact of these bioactive compounds on both clinical and commercial paradigms. This refined hypothesis encapsulates the scientific breadth of dietary polyphenols, linking their chemical diversity to a spectrum of biological and economic consequences.

**Figure 1:**
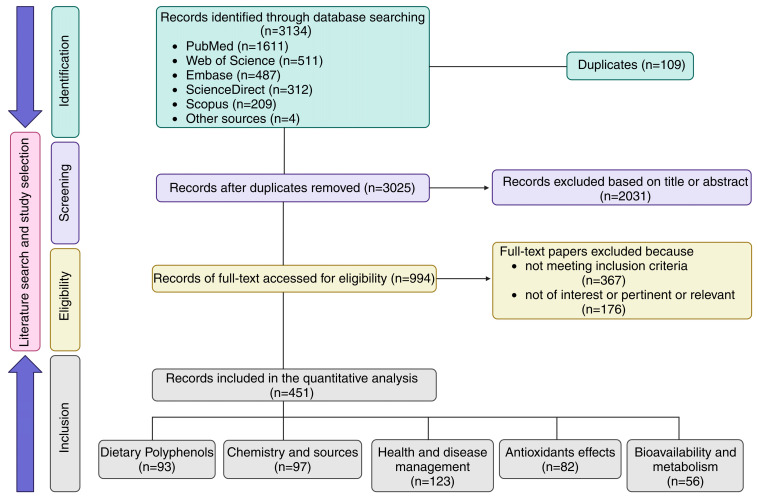
PRISMA diagram of literature search and study selection.

The overall process of literature search strategy and study selection was done according to PRISMA (Preferred reporting items for systematic review and meta-analysis) guidelines and is presented in Figure 1. The search terms and keywords for the study selection were dietary polyphenols OR food polyphenols AND chemistry and sources AND health and disease management AND antioxidant effects AND bioavailability and metabolism. The in vitro and in vivo (animal) studies along with pharmacological activity were used as further criteria for literature search. The search was carried out for last 40 years (1985 to 2024) of publications. Three independent reviewers (G.R., S.G. and S.C.) conducted the literature search in the scientific databases and assessed/verified the eligibility of the studies based on the title and abstract. Disagreement between reviewers was sorted out through consultation with fifth and sixth reviewers (M.R. and R.P.S.) to arrive at a consensus. The inclusion criteria were (i) studies involving dietary or food polyphenols and their role in health and disease management, (ii) studies reported on bioavailability and metabolism of dietary polyphenols, (iii) studies performed in polyphenol-rich extracts and their effects in in vitro or in vivo models, (iv) studies conducted on antioxidant role and pharmacological effects of health-promoting polyphenols, and (v) studies published from 1985 to December 2024 (40 years, both years included). The exclusion criteria were (i) studies involving dietary polyphenols intake as food supplements, food supplements and their role in disease management, (ii) studies on systematic reviews, meta-analysis and case reports, (iii) studies involving clinical data and human subjects, (iv) papers published before 1985, and (v) published articles in a language different from English. A total of 3134 published records were identified from the database search (PubMed Web of Science, Embase, ScienceDirect, Scopus) and other sources. After removing 109 duplicate articles, 3025 papers were screened and 2031 were excluded based on title and/or abstract. The full-text of eligible studies (n = 994) was read, 299 articles were excluded because not meeting the inclusion criteria (n = 367) or not of interest/pertinent/relevant (n = 176). At the end of the selection process, 451 papers were finally selected and included in the study.

## 2. Chemistry of Polyphenols

The classification of polyphenols is highly based on the topology of the phenolic ring and the side atoms or molecules attached to it [14]. Due to the varied topology and diverse availability, polyphenols are classified as follows:

### 2.1. Phenolic Acids

The phenolic structure with the carbon backbone of these acids consists of CO_2_H and OH group on the C_1_ and C_6_ carbon atoms [16]. They can either be hydroxybenzoic acids or hydroxycinnamic acids type, with a non-phenolic nature [12,17]. Some of the most common of this class are ferulic acid, sinapic acid, caffeic acid, chlorogenic acid, cinnamic acids, vanillic acid, gallic acid, syringic acid, protocatechuic acid, and coumaric acid [12]. The shikimate pathway from L-tyrosine has been reported as the biosynthetic source of phenolic acids [18], where biosynthetic sequences involving deamination [19], hydroxylation and methylation give rise to the acids [20].

### 2.2. Flavonoids

Structurally, they have the general backbone C6-C3-C6. Both C6 units (A and B ring) are phenolics [21]. Consequent upon the variation in the chromane ring (ring C), flavonoids have been classified as flavanones (hesperitin, naringenin, hesperidin, eriodyctiol), flavones (apigenin, tangeritin, baicalein, profilin), chalcones (phloretin, arbutin, phlorizin, chalco-naringenin), flavonols (quercetin, myricetin, rutin, morin, kaempferol), isoflavonoids (genistein, genistin, daidzein, daidzein), and anthocyanin (cyanidin, malvidin, delphinidin, peonidin [22,23,24]. C-glucosides and O-glucosides, integral subclasses of flavonoids, are characterized by a glucose molecule linked to a flavonoid backbone via a glycosidic bond, distinguishing them within the broader category of polyphenolic compounds found abundantly in plants. These glucosides are prevalent in various plant sources, including fruits, vegetables, grains, and herbs, contributing to their color, flavor, and nutritional profile. Exhibiting antioxidant, anti-inflammatory, and anticancer properties, C and O glucosides play crucial roles in human health by scavenging free radicals, modulating signaling pathways, and inhibiting enzymes involved in inflammation and carcinogenesis. Notable examples such as vitexin, isovitexin, quercetin-3-O-glucoside (isoquercetin), and kaempferol-3-O-glucoside (astragalin) exemplify the diversity and significance of these compounds in dietary sources, emphasizing their potential implications for human health and underscoring their importance in flavonoid chemistry [25,26].

### 2.3. Stilbenes

Stilbenes which are synthesized via the phenylpropanoid pathway is also a class of naturally occurring organic molecules distributed in fruits and other plant sources [27]. Structurally, they are made up of two aromatic rings (A and B) linked by a methylene bridge [28]. Some known stilbenes include resveratrol, piceatannol, pinosylvin, and rhapontigenin, among others [29]. Resveratrol is one of the most widely studied stilbene due to its wide range of pharmacological activities [28].

### 2.4. Tannins

These compounds have a molecular weight range between 500 to 30,000 Da and are distributed in various plant foods and beverages [30]. They can be either water soluble or insoluble, depending on their molecular weight. Based on their chemical structure, they are classified as condensed tannins, hydrolyzable tannins, phloro-tannins and complex tannins. The shikimate pathway is reported to be the biosynthetic route for the generation of tannins. Some of the known tannins include gallotannins, ellagitannins, proanthocyanidins, etc. [31].

### 2.5. Lignans

Lignans are formed by the union of two cinnamic acid molecules. Their structure consists of two benzylbutane, and are therefore called diphenolic compounds [32]. Lignans are low molecular weight phenolic compounds present in the cell walls of plants. They are plant components present in the glycosidic form. They provide rigidity to plant cells, facilitate mineral transport through vascular bundles, and protect plant cells from external injury. They are synthesized by the shikimic acid pathway in the plant cells [33].

### 2.6. Coumarins

Coumarins are a class of naturally occurring lactones composed of a benzopyrone framework [34]. The major classes of coumarins include simple coumarins, furocoumarins, dihydrofurocoumarins, pyranocoumarins (linear and angular), phenylcoumarins, and biscoumarins [35]. Mostly found coumarins are aesculin, aesculetin, umbelliferone, coumestrol, bergapten, psoralen etc. The shikimic acid pathway is the biosynthetic route for coumarins [36].

**Figure 2:**
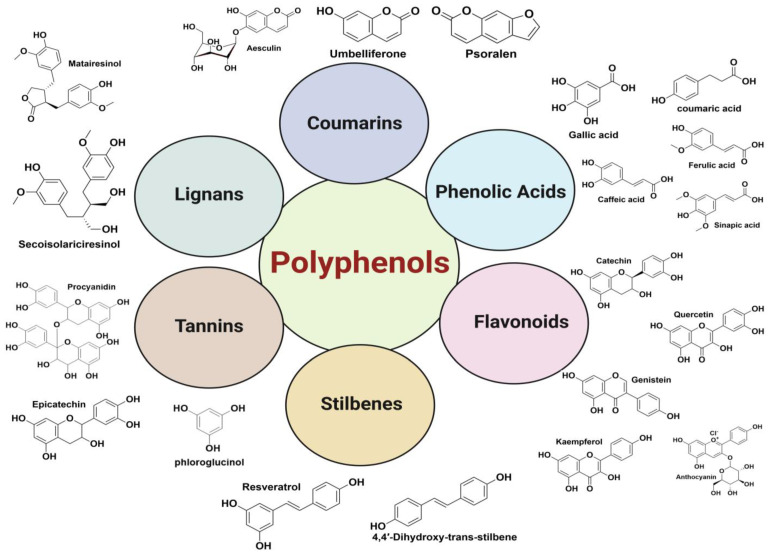
Chemistry of polyphenols.

The sub-classification of each of the polyphenols and structures has been mentioned in Table 1 and Figure 2, and few of them are discussed in the dietary sources, majorly because of their availability.

## 3. Dietary Sources

**Figure 3:**
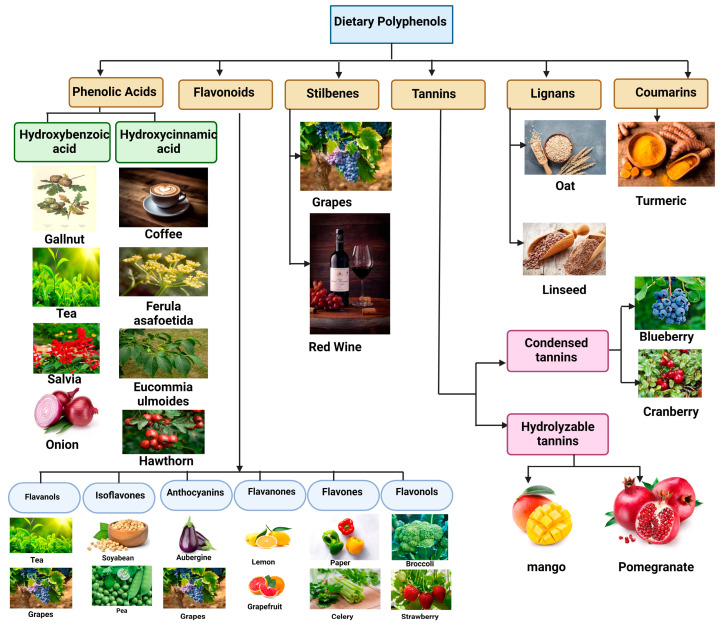
Various sources of dietary polyphenols.

According to recent research on their health advantages, it is essential to include polyphenols in the everyday diet [49]. The amount of each polyphenol is determined by the extraction process and in terms of approximate dry and fresh weight [50]. These compounds can be found in a variety of foods, such as berries, apples, citrus fruits, spinach, kale, broccoli, oats, whole grains, tea, coffee, and red wine, offering individuals numerous opportunities to incorporate polyphenols into their diets. Various sources of dietary polyphenols are displayed in Figure 3. The quantity of each polyphenol in these dietary sources can vary significantly, influenced by factors like the extraction process and the source material’s state, whether it is dry or fresh [50]. For example, the concentration of polyphenols may differ between fresh fruits and their dried counterparts, highlighting the importance of considering food processing methods when assessing polyphenol content. Factors like the specific variety and ripeness of fruits and vegetables can also impact polyphenol levels, further underscoring the complexity of estimating polyphenol intake from dietary sources, as shown in Table 2. Understanding the sources and approximate concentrations of polyphenols in foods is crucial for individuals seeking to optimize their dietary intake for health benefits. By incorporating a diverse array of polyphenol-rich foods into their diet, individuals can not only enhance flavor and culinary enjoyment but also naturally obtain these bioactive compounds, known for their antioxidant, anti-inflammatory, and potentially disease-preventive properties. Embracing a diet rich in polyphenol-containing foods can thus be a proactive step toward supporting overall health and well-being. The examples of polyphenols extracted as a dietary source as mentioned in Table 2 and are discussed in the section below.

### 3.1. Phenolic Acids

They are abundant in nature as herbs [51] and dietary [52] constituents, possess anti-oxidative potencies among others [53], and play a vital role in the control of diseases [54]. Mushrooms have been reported to be one of the major sources of phenolic acids, which substantially account for their therapeutic activities such as antioxidant, antimicrobial, and antitumor potencies [55]. Fruits and cereal grains are the abundant source of caffeic and ferulic acids [56].

### 3.2. Flavonoids

This is one of the classes of polyphenols that are widely distributed in vegetables, flowers, fruits, grains, wine, and tea [23]. They have variable polyphenolic structures and constitute the major components of polyphenolic compounds [57].

### 3.3. Stilbenes

Stilbenes are mainly found in *Vitis vinifera*, *Arachis hypogaea*, *Sorghum bicolor*, *Polygonum cuspidatum*, *Rhodomyrtus tomentosa*, *Rheum undulatum*, *Melaleuca leucadendron*, and *Euphorbia lagascae* [58]. They are among the class of compounds that give protection to plants against viral and other microbial-related attack [59,60]. Pinosylvin is present in high amounts in the heartwood of pine trees [61].

### 3.4. Tannins

Berries and their derivatives such as jams, jellies, and juices, are important sources of tannins [62]. They are responsible for imparting astringent and bitter taste to food products [31]. Due to their astringent property, they can alter protein structure, disrupt the activity of digestive enzymes, and form chelates with calcium and ferric ions, thereby reducing their bioavailability [31]. Epigallocatechin and epigallocatechin-3-gallate are the major tannins present in green tea [63].

### 3.5. Lignans

Dietary sources of lignans include rapeseed, flax seeds, sesame seeds, legumes, rye, barley, vegetables, fruits, fish, meat, etc. [64]. The common lignans present in almonds, cashew nuts, pecans, and other nuts are lariciresinol, matairesinol, secoisolariresinol, cyclolariciresinol, and 7-hydroxymatairesinol [65]. The lignans exert antioxidant, antiviral, and anticancer effects.

### 3.6. Coumarins

They are present in a variety of plant species belonging to the families of *Apiaceae* (*Umbelliferae*), *Rutaceae*, *Asteraceae* (*Compositae*), *Fabaceae* (*Leguminosae*), *Oleaceae*, *Moraceae*, and *Thymelaeaceae* [34]. Amongst them, the *Apiaceae* family is an important source of coumarins. The plants consisting of coumarins are used widely for culinary purposes and as flavoring agents in the food industry [66].

**Table 2:** Dietary sources of some polyphenols with their concentration in dry and fresh weight.

Polyphenol	Dietary Source	Concentration (~Dry/Fresh Weight)	References
Gallic acid	Black currant	7.67 to 39.70 mg/100 mg	[56,67]
Protocatechuic acid	Raspberry	215.28 mg/100 g	[68,69]
*p*-Hydroxybenzoic acid	Strawberry	2–8 mg/100 g	[68,70]
Caffeic acid	Kiwi	0.009–0.04 µg/g	[68,71]
Chlorogenic acid	Cherry	1–4 mg/100 g	[68,72,73]
Coumaric acid	Carrots	6.8 mg/100 g	[68,72,74]
Sinapic acid	Apple, Pear, Coffee etc.	0.140 mg/g	[68,72]
Anthocyanins	Aubergine	12.08 mg/100 g	[75,76]
Apigenin	Parsley, Chamomile	45 mg/g	[68]
Quercetin	Curly kale	8 mg/100 g	[77,78]
Kaempferol	Leek	32.5 mg/100 g	[77,78]
Myricetin	Broccoli, Red wine, Blueberry, Beans, Tomato, Black tea, etc.	3.8 to 22.6 mg/L	[79,80]
Epigenin	Celery	19.1 mg/100 g	[79,81]
Genistein	Soybeans	0.03–0.2 mg/100 g	[79]
Hesperidin	Grapefruit juice	0.93 mg/mL	[82,83]
Naringenin	Lemon juice	1.77 mg/100 mL	[82,83]
Catechin	Beans	8 to 12 mg/100 g	[84,85]
Epicatechin	Apricot, Cherry, Peach, Blackberry, Apple, Green tea, etc.	8.3 mg/100 g	[84,86]
Morin	Guava	*-*	[82]

## 4. Bioavailability and Metabolism

**Figure 4:**
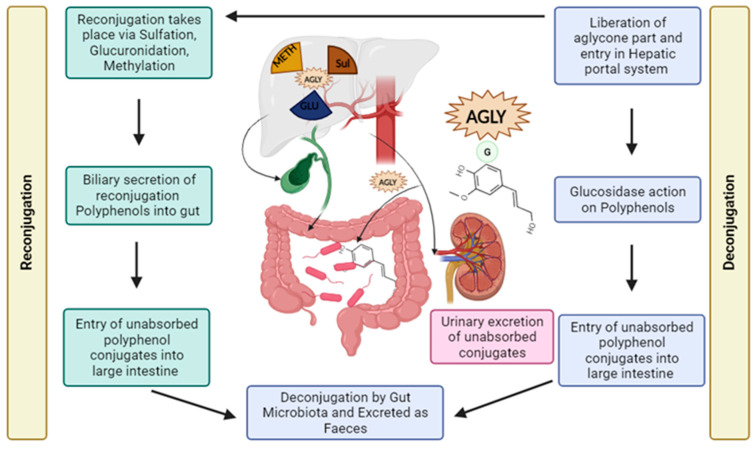
Uptake of dietary polyphenols (absorption) and their further metabolism.

Polyphenols post-consumption is digested in the stomach. In their native forms, polyphenols are present as conjugates with, majorly, a glycosidic side chain. These conjugations render the absorption of polyphenols by the small intestine rather difficult. Extracellular enzymes, such as glucosidases, help deconjugate the polyphenol, thus aiding in its absorption. Unabsorbed polyphenols are transferred to the large intestine where they undergo microbial modification to facilitate absorption. The deconjugated polyphenols are transferred to the liver via the hepato-portal vein, where most polyphenol metabolism occurs. Polyphenols are reconjugated in the liver with the help of various enzymes and make their way to the target sites. The liver also helps in excretion of excess polyphenols. Polyphenols are excreted in two major ways: urinary excretion by kidneys and biliary excretion, which are further processed and excreted finally as feces. The illustration is depicted in Figure 4 [87].

### 4.1. Phenolic Acids

The wide distribution of phenolic acids among plant-derived foods has been reported [88]. Coffee is one of the sources of phenolic acids that have been used to monitor the ADME (adsorption, distribution, metabolism and excretion) properties of phenolic acids [89]. The metabolic processes have been revealed to involve; methylation [90], glucuronidation [91], and sulfation [92], which takes place in the small intestine and subsequently in the liver [93], producing conjugates of these metabolic processes [94]. Such metabolites (glucuronidated, methylated, and sulfated phenolic acids) have been found in the biological fluids of patients who ingested coffee [95]. The presence of glucuronide and sulfate conjugates of hydroxycinnamic acids has been reported from the consumption of a particular wine drink [96]. Caffeic, ferulic, and chlorogenic acids are rapidly absorbed from the stomach and intestine in their free form; further, they are conjugated through detoxification enzymes [97]. Metabolism detoxifies the compounds by transforming them into an easily excretable form. However, some metabolites are therapeutically active in themselves [98]. The mode and site of metabolism depend on the phenolic acid’s chemical structure [99]. For instance, the colon is the only site of metabolism for chlorogenic acid [100]. Free phenolic acids are more easily absorbed than the corresponding esters [101].

### 4.2. Flavonoids

Due to the polyphenolic nature of flavonoid, it is commonly known for its antioxidant activity. Through flavonoid reaction with free radicals, reactive oxygen species are stabilized [102]. They prevent lipid peroxidation and the formation of superoxide via various pathways [103]. With the exception of flavan-3-ols, most flavonoids exist as glycosides [104]. Lower molecular weight proanthocyanidins have been reported to be better superoxide and hydroxyl radical scavengers, and xanthine oxidase inhibitors [105]. Among the class of flavonoids, it has been shown that isoflavones are the most absorbed while flavanol glycosides, flavanones, and flavonols have intermediate absorption. Anthocyanins, proanthocyanidins, and flavanol gallate were reported to have reduced absorption and bioavailability [106]. Kidney bean has recently been shown to have a lower bioavailability than two other (soya bean and faba bean) dietary legumes [107]. It has been revealed that the bioavailability of flavonoids is generally low. However, the bioavailability varies according to the class and molecular size of the flavonoids involved. Low bioavailability is found among high molecular weight flavonoids [108]. Conjugation is one of the metabolic processes found in flavonoids. Flavonoids undergo glucuronidation, sulfation, and methylation to form the corresponding esters, usually without aglycone in the plasma [109]. The small intestine is the first site (phase I metabolism) of flavonoid conjugation, and then the liver, where phase II metabolism occurs. Unabsorbed metabolites undergo further modifications in the microflora colon; the gut microbiota plays a vital role in metabolizing flavonoids, breaking them down into smaller, more bioactive compounds. Through processes like deglycosylation and ring cleavage, gut bacteria transform flavonoids into metabolites with enhanced health benefits. This microbial metabolism influences various physiological processes, such as antioxidant activity and inflammation modulation. The composition and activity of the gut microbiota vary between individuals, affecting the extent and pattern of flavonoid metabolism. Understanding this interaction is essential for harnessing the full therapeutic potential of flavonoids through dietary or microbiome-targeted interventions [110]. Glycosylation has also been noted as a major factor affecting bioavailability; quercetin glycosides were absorbed ten times faster than the corresponding rutinosides [111,112] in humans. This flavonoid possesses poor intestinal absorption and is only absorbed by the enterohepatic system when hydrolyzed into its constituents, quercetin and sugar moiety, by the microflora of the lower bowel. Results from an animal study using rats identified 3-methoxy-4-hydroxy phenylacetic acid, 3,4-dihydroxy phenylacetic acid, and m-hydroxy phenyl acetic acid as metabolites after oral administration of quercetin, while 3-*O*-methylquercetin has also been identified as of its metabolites from bile [113]. The bioavailability of quercetin has been reported to be low. This is the quantity of flavonoid that is present and unchanged in the systemic circulation [114]. The low bioavailability has been attributed to its high metabolism rate [115,116]. There are some reported flavonoids that undergo metabolic conversion in the enterocytes and liver [117], and such flavonoids would only have the metabolites found in the plasma [118]. Microemulsion, nano- delivery systems, microencapsulation and enzymatic methylation are some of the methods employed to combat the challenge posed by flavonoid bioavailability [119,120,121].

### 4.3. Stilbenes

Stilbenes also have a record of low bioavailability; however, they are distributed to various tissues in the form of glucuronides and sulfate conjugates in the tissues and plasma [122]. Among these, glucuronide has been found to be predominant as a metabolite, and its formation is more rapid [123]. The oral absorption of resveratrol in humans is found to be 75%; however, its bioavailability was found to be only 1% [124]. About 90% of resveratrol is subjected to fermentation by gut microbes. After absorption, the resveratrol conjugates enter the systemic circulation and penetrate the target tissues to execute their physiological action. In clinical trials it was found that a high rate of metabolic breakdown of resveratrol into resveratrol-3-*O*-sulfate, resveratrol-4′-*O*-glucuronide, and resveratrol-3-*O*-glucuronide limits its use in pharmacological applications [125,126]. Several research studies have been carried out to improve stilbenes’ bioavailability, solubility, and stability. The bioavailability of rhaponticin, a methylated derivative of resveratrol, was improved by modifying liposomes with polyethylene glycol. It was observed that its distribution was lowered in the gastrointestinal tract thereby enhancing the plasma concentration by 4.5 times [127]. Piceatannol, a monomer derivative of resveratrol, exhibited a higher absorption rate (maximum serum concentration was 2.6 times higher) and better metabolic stability (higher area under the curve) than resveratrol [128]. It was demonstrated that oxy-resveratrol was less bioavailable in comparison to resveratrol and underwent extensive hepatic metabolism to be excreted in bile and urine [129]. Hence, the bioavailability of the stilbenes largely depends on their structures.

### 4.4. Tannins

There are few reports on the bioavailability of tannins; however, it has been reported that procyanidins from apple juice gave a recovery of about 90% after consumption [130]. The bioavailability of tannins largely depends on factors such as chemical and biological degradation, gut and liver metabolism, membrane permeability and many more [131]. The degree of polymerization and solubility of tannins affects their rate of absorption, as highly polymerized tannins cannot be absorbed. Low solubility, formation of insoluble complexes and irreversible binding with DNA and proteins limit the absorption of ellagic acid [132]. In the small intestine, high molecular weight proanthocyanidins form complexes, thus interfering with their digestion. On the other hand, absorption of gallic acid from the small intestine takes place either by rapid permeation or in the form of conjugates [133]. The colonic bacteria help in the metabolism of hydrolyzable tannins. It is reported that ellagitannin is hydrolyzed by microbiota in the large intestine to ellagic acid and urolithin B [134]. Therefore, it is observed that metabolism and absorption of tannins take place at different parts of the gastrointestinal tract. Tannins have anti-nutritional properties and can retard the absorption of vitamins and minerals. They have also shown significant antioxidant activity [135]. Some studies report that tannins can exert pharmacological effects either as absorbable metabolite or as an un-absorbable complex [136].

### 4.5. Lignans

Lignans are metabolized into mammalian lignans by intestinal bacteria. Secoisolariciresinol diglucoside, a lignan present in flax seeds, is converted in the human colon to enterodiol, and entero-lactone exhibits anticancer effects [137]. The conversion of lignans to enterolignans largely depends on the activity of gut bacteria. The amount of precursor intake, gut microbial activity, and degree of conjugation determines enterolignan exposure. The lignans are conjugated as glucuronides or sulfates in the intestinal epithelium and liver; later, they are excreted in the urine and bile. The re-excreted compounds are then deconjugated by bacterial β-glucuronidase to undergo enterohepatic recycling [138]. The enterolignans take approximately 8–10 h to appear in the circulation after ingestion, while plant lignans take only 2 h [139].

### 4.6. Coumarins

These compounds are attributed to many pharmacological properties such as antioxidant, antimicrobial, anticoagulant, anti-inflammatory, anti-hypertensive, neuroprotective, etc. [140]. The pharmacokinetic profile of aesculin and aesculetin were studied in rats. After oral administration of aesculin (120 mg/kg), the C_max_ and AUC values were 340.3 ng/mL and 377.3 h ng/mL, respectively; on the other hand, for aesculetin it was found to be 316.5 ng/mL and 1276.5 h ng/mL, respectively. Further, the oral bioavailability of aesculin was found to be 0.62%, possibly due to poor oral absorption and first-pass metabolism [141]. In a non-compartmental analysis, the mean oral bioavailability of aesculetin was reported to be 19% [142]. The coumarin bergapten obtained an oral bioavailability of 69.5 ± 44.2% at a 15 mg/kg concentration and had good absorption from the gastrointestinal tract. However, its presence in the rat urine was detected only after 72 h [143].

## 5. Antioxidant Effects of Dietary Polyphenols

Epidemiological studies have shown that consumption of dietary polyphenols can reduce the risk of chronic diseases [10]. Phyto-antioxidants are the main contributors to the total antioxidant activity of fruits and vegetables [144]. They protect the cells from oxidative damage and increase the plasma antioxidant capacity [145].

The intricate relationship between flavonoids and the gut microbiota underscores the pivotal role of microbial metabolism in shaping the bioavailability and health effects of these polyphenolic compounds. Within the large intestine, flavonoids undergo extensive biotransformation by the diverse microbial community, yielding metabolites with potent physiological activities. Processes such as deglycosylation, ring cleavage, and demethylation lead to the generation of bioactive compounds that exert antioxidant, anti-inflammatory, and metabolic modulatory effects. This microbial metabolism enhances the bioavailability of flavonoids and influences their distribution and efficacy in various tissues. Understanding the dynamic interplay between flavonoids and gut microbiota holds promise for unlocking novel therapeutic strategies aimed at optimizing human health through dietary interventions and microbiome-targeted approaches [110,145].

Moreover, human health is greatly affected by the gut microbiota, and a case study was performed to comprehend the impact of polyphenols on it. According to the Ma et al., 2020 case study, the organisms provided with controlled administration of polyphenols were observed to have an abundance of Lactobacillus and Bifidobacterium and reduced levels of pathogenic *Clostridium* [49]. More studies related to anti-cancer properties have highlighted the positive effects of polyphenols. The antioxidant potential of polyphenols has been reported in various in vitro and in vivo models; a few are mentioned in Table 3 [146].

### 5.1. Phenolic Acids

In in vivo and in vitro studies, the antioxidant activity of chlorogenic and caffeic acid has been reported, where caffeic acid exhibited more antioxidant effects than chlorogenic acid. It was found that both compounds show protective effects against I/R injury, but the uptake of caffeic acid by Cao-2 cells was much more than that of chlorogenic acid [155]. The chlorogenic acid isomers, neochlorogenic acid and crypto chlorogenic acid present in *Prunus domestica* L. have been reported to exhibit antioxidant activities. Both the isomers showed superoxide radical scavenging activity and inhibited oxidation of methyl linoleate [156]. This scavenging effect of crypto chlorogenic acid, which is also a major component of mulberry leaf, is responsible for its protective effect on β-cells function in diabetes [157]. In another study, the three isomers of caffeoylquinic acid, namely 3-*O*-caffeoylquinic acid, 4-*O*-caffeoylquinic acid, and 5-*O*-caffeoylquinic acid, with three isomers of di caffeoylquinic acid namely, 3,5-dicaffeoyl-quinic acid, 3,4-dicaffeoylquinic acid, and 4,5-dicaffeoylquinic acid exhibited antioxidant potency alongside protected DNA from damage [158]. However, the dicaffeoylquinic acid isomers exhibited a better antioxidant effect as they contain more hydroxyl groups. On the contrary, the corresponding caffeoyl-quinic acid isomers exhibited similar antioxidant effects [159].

Further, chlorogenic acid, alongside its associated moieties, caffeic and quinic acids, has been reported to significantly act against rat hepatoma cell line (AH109) [160]. However, they were observed not to affect the proliferation of the hepatoma. A structure-activity relationship observation depicts that caffeic and quinic acid confers an additive inhibitory potency on chlorogenic acid following the suppression percentage recorded for each [161]. The observations also suggested that the 3,4-dihydroxy group in caffeic acid is partly responsible for its anti-invasive activity [162]. Additionally, in the carrageenan-induced [163] inflammation model, oral administration of chlorogenic acid resulted in activity that was comparable to indomethacin, the reference drug [164]. El-khadragy and colleagues revealed in recent research that chlorogenic acid, through its antioxidant properties, improved the testicular damage induced by arsenic [165]. Also, chlorogenic acid has been revealed to possess anti-obesity [166], antidiabetic [167], antimicrobial [168], and antihypertensive [169] effects. The antidiabetic activity of this compound has been linked to its involvement with glucose metabolism [170].

Furthermore, YES-10^®^ is a combination therapy of *Erigeron annus* (L.). PERS. and *Clematis mandshurica* RUPR. (CMR) leaves which are rich in chlorogenic acid and scutellarin [171] have been reported to increase superoxide dismutase (SODs) in CA1 pyramidal neurons [172]. It also protected the CA1 from TI injury. This particular feature has proposed YES-10^®^ as a candidate for the development of drug to guard the brain against ischemic damage [173].

Sinapic acid and the corresponding alkyl esters are among the phenolic acids that have exhibited considerable antioxidant effects. Experimental reports from DPPH and FRAP assay suggest that sinapic acid has a higher activity than the corresponding alkyl esters [174]. This polyphenolic acid is present in coffee [175], vegetables [176] and cereal grains. An isolated sinapic ester derivative, 6-O-sinapoyl sucrose was characterized on isolation and revealed antioxidant activity using DPPH free radical scavenging test [177]. A range of sinapic acid derivatives found in rapeseed have been shown to possess antioxidant potencies [178]. The anti-inflammatory, anticancer, neuroprotective, antimutagenic, and antiglycemic activities of the sinapic acid derivatives have been attributed to their antioxidant potencies [179]. Sinapic acid has been described to attenuate high blood pressure, vascular dysfunction, cardiac fibrosis and myocardial by preventing oxidative stress through its antioxidative potency [180]. SH-SY5Y is one of the human-derived cells employed in neurodegenerative-associated research [181]. More recently, it has been shown to defend SH-SY5Y human neuroblastoma cells against 6-hydroxy dopamine-induced neurotoxicity. It was achieved through a significant oxidative blockage and reactive oxygen species (ROS) attenuation overproduction [182].

Ferulic acid, over the years, has been known for its antioxidant activities and stands as a major feature of why it has been a choice as cosmetic and food additives and is well found and indispensable in other industrial applications [183]. This class of phenolics has been described as the most effective among other phenolic acids whose scavenging potency was tested by Kikuzaki and colleagues [184]. As a result of the antioxidant potencies of phenolic acids, this class of compounds has also exhibited antioxidant-related activities [185]. Ferulic acid has been found to reduce memory dysfunction while exerting protective effects against oxidative stress as well as apoptosis resulting from IRI injury [186] through the inhibition of the TLR4/MYD83 signaling pathway [187]. This activity results from the activation of the P38 MAPK-mediated signal cascade [188]. The promotion of functional recovery from ischemic injury induced by middle cerebral artery occlusion in rats by an extract from rice is attributed to its ferulic acid component [189]. It also inhibits nitric oxide synthase proteins [190]. Solid-state fermentation has been used to release ferulic acid in wheat bran which exhibited a better scavenging effect than the free ferulic acid [191]. Kelainy et al. investigated the activity of ferulic acid against lead-induced oxidative stress [192]. In this study, animals were orally administered with lead acetate for 10 days at a dose of 20 mg/kg. Oxidative stress was observed in the form of excess production of LPO, ROS, MDA, and DNA damage alongside reduced levels of sex hormones [193]. Animals treated with ferulic acid exhibited no DNA damage such as DNA apoptotic fragmentation, compared to the control group. Generally, the observation recorded for ferulic acid-treated animals revealed that the compound possesses a protective activity against lead acetate-induced oxidative stress [194]. The result of the study conducted by Wang et al. revealed the antioxidative potency of ferulic acid and its ability to induce lipid metabolism in weaned piglets through the usage of dietary ferulic acid supplementation [195]. Oral administration of ferulic acid or ethyl ferulate has been proved under experimental investigation to cause retinal protective effect in a sodium iodate-induced model of retinal degeneration [196] in human retinal pigment epithelial cell lines. It has also reduced sodium iodate-induced retinal degeneration’s morphological and functional features [197]. An in vitro study using a set of biophysical methods has revealed the protective function of ferulic acid on amyloid formation of bovine β-lactoglobulin, which is associated with neurodegenerative disorders among other diseases such as systemic amyloidosis [198]. Ferulic acid and ferulic derivatives synthesized by Lambruschini and colleagues were reported to possess a protective potency of human endothelial cord vein cells against the oxidative damage of hydrogen peroxide [199]. The use of supplements containing this polyphenol in oxidized fish oil-induced oxidative stress, has been reported to cause antioxidative effects in tilapia [200]. It has also been shown to have a neuroprotective effect on amyloid-beta (Aβ) neurotoxicity-induced Alzheimer’s disease (AD) [201].

The cinnamic acid present in *Barringtonia asiatica* stem-bark has also been reported to have an antioxidant effect and antifungal activity against *Aspergillus niger*, *Fusarium oxysporium*, *Candida tropicalis*, and *Aspergillus flavin* [202]. Among the 23 cinnamic ester derivatives [203] screened against *C. albicans* strains, only methyl caffeate and methyl 2-nitro cinnamate exhibited the largest antifungal activity [204]. Various cinnamic acid derivatives have recently been reported to possess anticancer, anti-TB, and antimalarial activities [205]. Protocatechuic acid, due to its antioxidant properties, has been found to be beneficial in cancer, ulcer, fibrosis [206], inflammation, [207] atherosclerosis, and viral infections [208]. It also protects the cardiac and kidneys from oxidative stress [209].

Gallic acid and its derivatives have been reported for their antioxidant properties. They scavenged hypochlorous acid. Gallic acid lauryl ester in ethanol reduced the peroxidation of ox brain phospholipids. However, gallic acid, its methyl ester and propyl gallate have exhibited a prooxidative effect by enhancing sugar deoxyribose damage in the presence of hydrogen peroxide and ferric-EDTA [210,211]. The reduction of ethanol-induced gastric ulcers by gallic acid in rats has been associated with the Nrf2/HO-1 antioxidative signaling pathway and anti-apoptosis function [212]. Its antioxidant defense mechanism has exhibited a protective effect against cadmium chloride-induced alterations in Wistar rats [213].

### 5.2. Flavonoids

Many flavonoids have been reported for their antioxidant activity [214]. Pekkarinen and coworkers have reported the antioxidant activity of quercetin, myricetin, kaempferol, and (+)-catechin in methyl linoleate [215]. All the flavonoids were observed to have inhibited the formation of hydrogen peroxides in methyl linoleate, although kaempferol alongside rutin exhibited a relatively weak antioxidative response, while quercetin and myricetin showed a better inhibition in comparison to α-tocopherol [215]. The exceptional antioxidant potency of quercetin has been attributed to the presence and structural nature of the C-ring. The double bond between carbon 2 and 3 in the C-ring and the presence of the keto group in C4 are reportedly responsible for the antioxidant potencies of quercetin [216]. The absence of a hydroxyl group at the C3 which is present in quercetin and (+)-catechin accounts for the relatively low antioxidative effects of rutin. It has also been proven in this study that the antioxidant activity of flavonoids is independent of their solubility, as the more hydrophobic quercetin had a better hydroperoxides formation inhibition than the hydrophilic glycoside rutin. Hence, their antioxidant activity was attributed to both the presence of the C3 hydroxyl group, and the metal chelation potency of the complex formed by the C4 and C3 keto group and hydroxyl group, respectively [217]. In another structural activity relationship report, it was revealed that 3^1^, 4^1^-dihydroxy catechin in the β-ring, 2,3 unsaturated alongside the oxo functional group at the position four in the C-ring of the flavonoids, hyperoxides, and myricetin have been attributed to the antioxidant potency of the flavonoid [218]. It has been noted that the free radical scavenging effect of flavonoids is a result of the formation of less reactive phenoxyl radicals through electron or hydrogen donation, hence inhibiting the hydrogen peroxide-driven Fenton reaction [219]. Their chelating ability has been reported as a consequence of the 4-carbonyl, 5-hydroxyl, and catechol groups on the β-ring [220].

Among six quercetin derivatives that were collectively studied for their antioxidant activity using the in vitro method by DPPH and FRAP assay, quercetin, tamarixetin, isorhamnetin, and quercetin-3-*O*-glucuronide exhibited significant antioxidant potency [221]. Quercetin acts mechanistically in various ways towards preventing oxidative stress. It inhibits the activation of NF-κB and MAPK signaling pathways alongside the expression of apoptosis-related proteins brought about by LPS/d-GUIN [222], enhances Nrf2/GSH antioxidant signal transduction pathways [223], regulation of phosphoinositide-3-kinasepathway [224]. Other mechanisms include the regulation of TLR2 signaling pathways [225], and the protection of BEAS-2B cells from Cr (VI) induction through targeting of miR-21-PDCD4 signaling [226]. Quercetin also enhances Nrf2-ARE signaling [227] and controls the transcription activities of NF-κB and AP-1 [228]. In order to improve the bioavailability of quercetin, modification to the parent structure has been performed [229] through derivatization and recombination with other active groups [230]. Quercetin metallic complexes have also been shown to possess better antioxidant activity than quercetin, the parent compound [231]. Three quercetin cyclodextrin complexes, β-cyclodextrin, sulfobutyl ether-β-cyclodextrin, and hydroxy propyl-β-cyclodextrin, showed good reactivity with DPPH [232]. Copper complexed quercetin has been evaluated using DPPH. The complex exhibited better activity than the free flavonoid [233]. Wu and colleagues have demonstrated in their research that quercetin-loaded nanoparticles prepared using the nanoprecipitation method exhibit more antioxidant activity than the pure drug [234]. Some metallic complexes of quercetin have also shown good antioxidant potency. The DPPH scavenging potency of the quercetin copper complex is more than that of free flavonoids [235]. However, another study revealed that the scavenging activity of free quercetin decreased after being chelated with tin (II) ion [236]. A study on the determination of quercetin in the plasma of healthy individuals (volunteers) after intake of flavonoid-containing meals revealed that 3^1^ position methylated, sulfate, and glucuronic acid conjugates as the major metabolites of quercetin [237]. Quercetin-cadmium complex has also shown a lesser antioxidant activity than the free quercetin [238]. Quercetin-DNA complex has exhibited a stronger free radical scavenging and antioxidant potency than free quercetin both experimentally and computationally [239]. In order to improve the cellular penetration and antioxidant potency of quercetin, nanoparticles of titanium dioxide in a study have been conjugated with quercetin. The result revealed that the quercetin–nanoparticle conjugate enhances a high quercetin bioavailability and stability while exhibiting antioxidant potency against reactive oxygen species (ROS) without observable toxicity [240]. The antihyperglycemic effect of quercetin in fructose-streptozotocin-induced diabetic rats has been attributed to the flavonoid’s ability to improve the pancreatic antioxidant status [241]. Copper (II) complexes of quercetin and curcumin have been synthesized, characterized, and investigated for their biological activity. Both complexes exhibited significant scavenging activity with no observable toxicity in the eukaryotic experimental model of *Saccharomyces cerevisiae* [242]. Quercetin and naringenin use antioxidant mechanisms to protect stored boar semen. In research where this was investigated, it was revealed that while naringenin attenuated ROS concentration, quercetin was potent in quenching superoxide, and both molecules have been demonstrated to be potential semen enhancement supplements [243]. The human serum albumin nano-complex of quercetin attenuates hydrogen peroxide-induced neuron mortality through an observable increase in the action of SOD and CAT. The formation of the complex also improved the bioavailability of quercetin [244].

Kaempferol is another naturally occurring flavonoid with known antioxidant potency. It has been reported to act against rotenone-induced Parkinson’s disease (PD) by increasing antioxidant makers in the PD model and SH-SY5Y cells [245]. Aside from the catalytic and anticancer activity of gold nanoparticles synthesized using kaempferol glucoside, the nano complex also exhibited a higher radical scavenging activity when compared to the flavonoid extract [246]. This flavonoid stimulates NF-κB signaling, down-regulates interleukin-6 (IL-6). It also increases the mRNA expression of IL-β, TNF-α, and NF-κB1 [247]. Kaempferol derivatives isolated from *Bryophyllum pinnatum* exhibited antioxidant and antimicrobial activity. Among the derivatives, the α-rhamnoisorobin derivative demonstrated the highest activity [248]. Kaempferol has been reported to exhibit NO-release T-cell proliferation compared to its glycosides, thereby possessing a better antioxidant activity [249].

Hesperidin, a flavonoid in sweet orange and lemon, is also known for its various biological effects [250]. It has been shown to possess a DPPH scavenging effect [251]. However, aglycone hesperetin demonstrated better antioxidant and neuroprotective activity than the parent compound and thus has been suggested to be a promising drug potential in the treatment and prevention of neurodegenerative disorders [252]. Superoxide dismutase-like activity and antiproliferative activities of hesperidin were improved when complexed with vanadium metal [253]. Hesperidin decreased level of marker enzymes and improved the antioxidant status in nicotine-induced lung toxicity in rats [254]. Mechanistically, hesperidin exhibits antioxidant activities through the inhibition of nitric oxide radicals, lipid peroxidation, attenuation of TBARS, and hydroxy radical inhibition [255]. It also ameliorates spinal cord injury-induced motor dysfunction and neuro-pathological degeneration in rat models through mechanistic effects via the Nrf-2/HO-1 pathway [256].

The research conducted by Wang’s research group on the antioxidant effect of myricetin on oxidative stress–induced cell damage has suggested that myricetin has the potency to inhibit the reactive oxygen species and oxidation-induced adverse effect, thereby protecting the cellular environment from free radical damage while inducing the cellular antioxidant enzyme defense system and repairs the damage of DNA and lipid [257]. A computational study has suggested that myricetin derivatives, 3,4-di-*O*-alpha-L-rhamnopyranoside are potential antioxidants [258]. Myricetin has been reported to act against other cell oxidation-related disease such as inflammation, diabetes, epilepsy, cardiac disease, cancer and Alzheimer’s disease [259]. Recently, myricetin has been reported to have protected the lymphocytes of healthy individuals and pre-cancerous MGUS patients against hydrogen peroxide induced reactive oxygen species related oxidative damage [260].

Isorhamnetin isolated from the sea buck thorn marc has shown to possess a scavenging activity on DPPH radical, reducing Fe^3+^ to Fe^2+^ and chelates iron [261]. The antioxidant potency of this flavonoid has been attributed to its experimentally verified ability of enhancing cortico-hippo campal learning and memory capability in scopolamine induced amnesia in rats by increasing glutathione level, catalase and superoxide activities [262]. Isorhamnetin activates Nrf2-ARE pathway through phosphorylation of ERK1/2, PKCo and AMPK in hepatocytes. It also inhibits ROS production [263]. It has been used to attenuate and treat neurodegenerative disorders [264]. Due to its ability to inhibit the stability of Aβ aggregates thereby prevents cells against Aβ-induced cytotoxicity among other related activities. Isorhamnetin has been described as having the potential of preventing the initiation of Alzheimer’s diseases [265]. Among other highlighted mode of actions, the flavonoid has been reported to activate PBK/AKT signal transduction pathway, activates Nrf2/ARE, Nrf2/HO-1 and ERK pathways and thus inhibits apoptosis, protect cells from peroxide damage [266]. It also induces cell death through ERK/MAPK and ROS pathways in OSCC cells [267]. A comparative study revealed that naringenin has a higher antioxidant capacity than the corresponding glycoside [268].

Naringenin is one of the flavonoids widely distributed in fruits, tomatoes, and citrus fruits [269] with several reported antioxidant, anticancer, anti-inflammatory, neuroprotective, and cardioprotective activities [270]. Oxidative stress and toxicity induced by Lambda-cyhalothrin, a synthetic insecticide in the liver of male rats has been shown to be inhibited by naringenin [271]. This was achieved via modulation of oxidative-nitrosative stress, MMP-9, and cytokine levels. It has been shown to ameliorate diabetic neuropathic pain [272]. It possesses a protective ability of Aβ-induced neuronal cytotoxicity (in vitro) [273]. This flavonoid and its synthetic derivatives have demonstrated the ability to affect antioxidant enzyme activities of erythrocytes and liver in higher cholesterol-fed rats [274]. Baki and colleagues synthesized, characterized and assayed naringenin oxime for its antioxidant activity. The result of the study showed that the derivative exhibited an antioxidant potency higher than the parent compound [275]. Naringenin, curcumin and quercetin were reported to have improved memory retention, learning acquisition and also prevented memory extinction. Through the enhancement of antioxidant concentration and activities, naringenin and quercetin prevent the alteration of the brain’s antioxidant defense system [276]. Y(III) and EU(IV) complexes of naringenin-2-hydroxy benzoyl hydrazone were observed to have an active scavenging effect on OH [277]. It potentiates endogenous antioxidant status during hyperglycemia and attenuates the over-production of nitric oxide-induced inflammation [278]. In regulating doxorubicin–induced liver dysfunction, naringenin has been reported to inhibit ROS-induced lipid peroxidation, ROS production, prevent the reduction of the antioxidant armory; catalase, glutathione, reductase, superoxide dismutase (SOD) glutathione and peroxide (GPx) [279]. This flavonoid has also attenuated neuronal apoptosis in MG-treated NSC34 cells, hence improving antioxidant defense [280].

Apigenin is a naturally occurring flavonoid in vegetables, fruits and medicinal plants. It has a wide-reported range of antioxidant activities [281]. Apigenin has been revealed to act against the oxidative stress induced by carcinogens through attenuation of lipid peroxidation in N-nitroso-diethylamine-induced and phenobarbital-enhanced hepatocellular carcinogenesis [282]. Through modulation of pro-inflammatory cytokines and antioxidant activity, apigenin induced observable change in the level of pituitary-ovarian axis hormones in polycystic ovary syndrome in rat models [283]. In methotrexate–induced hepatotoxicity, apigenin caused alteration in antioxidant, inflammation and lipid peroxidation factors [284]. In a cellular oxidant defense, the flavonoid has inhibited streptozotocin-induced pancreatic β-cell damage [285]. Attenuation of circulating oxidants (LPO, OH) and improvement in the levels SOD, CAT, and GPx, as well as non-enzymic antioxidants have been attributed to luteolin in an azoxymethane-induced colon carcinogenesis [286].

Various studies that have shown Fisetin has antioxidant activity [287]. This antioxidant potential is also attributed to its neuroprotective, anti-inflammatory, and anticarcinogenic effects [288]. Quantum chemical-based calculations have shown that the 3-OH moiety of fisetin possesses the lowest bond dissociation energy followed by the 3,4-OH groups which account for its ability to donate hydrogen to potential free radicals. The B and C rings have been suggested to be more efficient in their antioxidative potencies than the A ring [289]. Bidya and colleagues demonstrated fisetin’s ameliorative effect on cisplatin-induced nephrotoxicity in rats through antioxidant defense and modulation of nuclear factor-factor kappa β (NF-κB) activation [290]. Reportedly, fisetin improves rotenone–induced motor impairments significantly. It has been hypothetically stated that fisetin could enhance mitochondrial enzyme activity, hence attenuating oxidative stress [291]. Fisetin has been observed to inhibit the potentiation of cell death by iron and copper in a therapeutic attempt to lower glutathione levels [292]. Oxidative stress and neuroinflammation induced by D-galactose have reportedly been ameliorated by fisetin in mice brains through the regulation of endogenous antioxidant mechanisms. It suppresses the activated P-JNK/NF-κB pathway [293] alongside the downstream targets and controls endogenous antioxidant mechanisms, hence inhibiting the accumulation of reactive oxygen species (ROS) [294]. This flavonoid enhances the expression of the antioxidant PON 2 through the activation of PPARY, attenuating vascular smooth muscle cell migration and proliferation and ameliorating neointimal hyperplasia after intimal injury [295]. Recently, the anticancer activity of fisetin alongside kaempferol has been attributed to their antioxidant activities [296]. It has been proven to inhibit proinflammatory makers such as NO, iNOS, IL1-β and TNF-α [297]. Through the activation of the reperfusion injury salvage kinase signaling pathway, fisetin has reportedly reduced myocardial ischemia [298]. Fibromyalgia induced by reserpine has been attenuated by fisetin through ROS and serotonergic pathway modulation [299]. The oral bioavailability of fisetin has been reported to enhance its neuroprotective activity in rat models of rotenone-induced Parkinson’s disease [300].

Three of the most common anthocyanidins, cyanidin, delphinidin, and malvidin have been predicted to have a radical scavenging ability using an ab initio computational study [301]. Malvidin has attenuated lipopolysaccharide-induced mitogen-activated protein kinase activation, and mitochondrial depolarization, among other oxidative factors [302]. It has been suggested that malvidin is vital in oxidative and inflammatory stress induced by the THP-1 cell line [303]. Delphinidin has attenuated the oxidative stress induced by hydrogen peroxide in HePG2 cells [304].

### 5.3. Stilbenes

The antioxidant activity of four stilbenes was evaluated by ORAC assay, where resveratrol exhibited the highest activity, followed by oxyresveratrol, pterostilbene, and pinosylvin. While in the ABTS assay, highest activity was shown by oxyresveratrol, followed by resveratrol, pinosylvin and pterostilbene [305].

Resveratrol has shown oxidative properties through lipid oxidation [306], prevention of DNA damage [307], inhibition of NF-κB activation, increasing the SOD activity, decreasing urinary8-hydroxy-2′-deoxy guanosine, activation of Nrf2 [308]. Resveratrol reduces the risk of cardiovascular disease by preventing lipid peroxidation [309]. It further prevents the oxidation of polyunsaturated fatty acids and is found to have stronger antioxidant activity than α-tocopherol [310]. It up regulates the expression of manganese superoxide dismutase (Mn-SOD) in myoblast cells through a mechanism dependent upon the nuclear factor (erythroid-derived 2)-like 2 (NRF2) thereby reducing the risk of hypertension [308]. Another study demonstrated that administration of resveratrol increases the activity of SOD and catalase [311]. Resveratrol exerts neuroprotective functions by up-regulating the endogenous antioxidant enzymes, inhibiting modulated NF-κB and peroxisome proliferator-activated receptors alpha (PPARα) [312,313]. In addition, due to its antioxidant properties, it counteracts the Aβ toxicity in AD patients [314]. The antidiabetic and anti-inflammatory activity of resveratrol is mainly attributed to its ability to reduce oxidative damage, reduce pro-inflammatory cytokines, and inhibit apoptosis [315]. It also prevents the development and progression of cancer by modulating the cellular pathways involved in inflammation, apoptosis, cell proliferation, metastasis etc. [316]. Similarly, oxyresveratrol showed inhibitory activity against DPPH, hydroxyl, nitric oxide, and hydrogen peroxide radicals [317]. It exerts neuroprotective activity by modulating MAPK and NF-κB signaling pathways [318]. When rat cortical neurons treated with amyloid β protein, were exposed to oxyresveratrol, it reduced ROS production, and glutamate release and suppressed the calcium levels in the cytoplasm, indicating a potential candidate in the treatment of AD [319]. However, this compound showed weak cytotoxicity against various cancer cell lines [320].

In a study, the compound piceatannol (200 mg/kg) showed better antioxidant activity than BHT (200 mg/kg), a synthetic antioxidant used in the preservation of food products. The higher antioxidant potential of piceatannol might be due to the presence of more hydroxyl groups than BHT [321]. Later, the protective effects of piceatannol on lipopolysaccharide (LPS) insult in mouse brain endothelial cell line (bEnd.3) was examined. The results of the study showed that piceatannol upregulated the expression of adhesion molecules (ICAM-1 and VCAM-1) and iNOS in LPS-treated bEnd.3 cells, which suggests its anti-inflammatory and antioxidant activity [322]. In addition, through its antioxidant mechanism piceatannol protected rat cardiomyocyte (H9c2) cells, normal human lung fibroblasts [152] cells, mouse lymphocytic leukemia (L1210) cells, human leukemia (K562) cells, and human promyelocytic leukemia (HL-60) cells from hydrogen peroxide-mediated cytotoxicity [323]. This stilbene provides protection against oxidative stress and inflammation to human retinal pigment epithelial (ARPE-19) cells through induction of heme oxygenase-1 (HO-1) enzyme [324].

Pinosylvin reduced the neutrophil count and decreased the concentration of oxidants for adjuvant arthritis both in the in vitro and in vivo rat models [325]. Another study demonstrated that pinosylvin given to adjuvant arthritic rats at a per oral dose of 50mg/kg improved NF-κB activity in the liver and lung, HO-1 expression and LOX activity in the lung, MCP-1 and F2-isoprostanes levels in the plasma [326].

Rhaponticin and its aglycone form, rhapontigenin inhibits the modulated NF-κB. The presence of oxygen-containing functional groups such as –OH and –OCH_3_ in the benzene ring might be responsible for their activity [327]. Further rhapontigenin up regulated the expression of SIRT1 in the THP-1 human monocytic cell line [328] which in turn inhibited the activity of NF-κB [329].

### 5.4. Tannins

The antioxidant potency of green tea has been attributed to one of the major phytoconstituents, catechin, which is widely distributed in green leaves [330]. The antioxidant of this flavonoid has been attributed to various molecular structural features. 1,4-pyrone moiety, alongside the 3-OH group has been described to affect the antioxidant potency of luteolin and catechin [331]. In comparing the metabolites of catechin and epicatechin with the parent compound, it was observed that the cleavage of the C-ring alongside α and β-oxidation increases their antioxidant activity. Among the metabolites, 1-(3′,4′-dihydroxyphenyl)-3-(2″,4″,6″-trihydroxy phenyl) propan-2-ol showed a high antioxidant activity twice as much as catechin, while the highest antioxidant activity was reported for 2-(3′,4′-dihydroxy phenyl) acetic acid whose DPPH and ABTS free scavenging activity equals that of the parent compound while its reducing ability is reported to be significantly higher than both catechin and epicatechin [332]. A density functional theory (DFT) study has suggested that the addition of N(CH_3_)_2_, an electron-donating group enhances the antioxidant potency of catechin [333]. The catechin derivative, catechin-5-O-gallate, exhibits antioxidant activity [334]. The antioxidant potency of *Tinospora cordifolia* has been attributed to the presence of epicatechin in the stem plant [335]. Some antioxidative attributes have been made for the bark extract of the Japanese knot weed rhizome, which contains (−)-epicatechin [336]. (−)-Epicatechin-3-gallate (EGCG), a major anticancer molecule in tea significantly scavenges OH radicals and inhibits the activation of NF-κB. The NF-κB was induced by Cr (IV) and 12-O-tetradecoryl-phorbol-13-acetate (TPA) [337]. In order to improve the lipid membrane permeability of EGCG, a novel lipophilic EGCG derivative, monoalkylated EGCG has been recently synthesized. However, its antioxidant activity decreased against DPPH free radicals, and cellular experiments suggest that the lipid moiety improves the antioxidant capacity of the EGCG derivative [338].

Corilagen, an ellagitannin, induces protective effects in cerebral ischemic injury by inhibiting oxidative stress and promoting angiogenesis by activating the Nrf2 signaling pathway [339]. Such a protective effect has also been recorded for this polyphenol for renal calcium oxalate crystal-induced oxidative stress apoptosis and inflammatory effect through the P13K/Akt and PPAR-γ pathways [340]. In N9 murine microglia cells, it has attenuated oxidative effects in tert-butyl hydroperoxide-induced oxidative stress [341]. The hepatoprotective and antioxidative properties of *Terminalia catappa* L. have been attributed to the corilagen, one of its bioactive constituents [342]. It reduces sleep deprivation-induced memory impairments through the regulation of NOX2 and Nrf2 activating factors [343]. It also affects MAPK and NF-κB signaling pathways and thus has been reported to ameliorate acetaminophen-induced hepatotoxicity in mouse models [344].

### 5.5. Lignans

In anin vitrosystem, secoisolariciresinol diglycoside and its mammalian lignan metabolites, enterodiol and enterolactone, inhibited the linoleic acid peroxidation at a concentration of 10 µM [345]. Further, enterolactone exhibited a significant cytotoxic effect in acute myeloid leukemia cells by enhancing DNA fragmentation and the intrinsic apoptotic pathway [346]. Another study demonstrated that dihydroguayaretic acid, guayacasin and isopregomisin are powerful antioxidants with activities similar to that of propyl gallate [347]. The antioxidant activity of schisandrene was evaluated by 2′,7′-dichlorodihydrofluorescein diacetate (DCFH-DA) cellular-based assay. A study on the structure-activity relationship suggested that the presence of exocyclic methylene functionality was responsible for antioxidant activity [348]. The flaxseed lignin, secoisolariciresinol diglucoside, enterolactone, and enterodiol exert antioxidant activity against DNA damage and lipid peroxidation and, therefore, were found to be beneficial in cancer, hypercholesterolemia, hyperglycemia, atherosclerosis, and lupus nephritis. In preclinical anticancer models, lignans reduced growth, progression, and angiogenesis [349]. Further, neolignan, isolariciresinol, and isolariciresinol isolated from German Riesling wine were reported to show antioxidant activity [350]. The sesame lignans, sesamol, sesamin and sesamolin showed lower antioxidant activity than tocols (α- and γ-tocopherols and α-tocotrienol) and butylated hydroxytoluene (BHT) in rat liver microsomes and cumene hydroperoxide (CumOOH)/Fe^2+^-ADP-NADPH (enzymatic) system [351].

### 5.6. Coumarins

Umbelliferone shows antioxidant activity in both in vitro and in vivo models. In the DPPH radical scavenging assay, it exhibited 59.6% inhibition in comparison to the standard drug which showed 96% inhibition [352]. In a site-specific deoxyribose degradation assay, it was reported that umbelliferone inhibited membrane reactive free hydroxyl radical by 63.6% [353]. Further, in ABTS scavenging and ferric reducing assay it had significant reducing capacity and had higher antiradical power than ascorbic acid [354]. Later, in irradiated lymphocytes, this compound not only reduced intracellular reactive oxygen species levels but also restored mitochondrial membrane potential and prevented DNA damage [355]. Umbelliferone also shows strong antinociceptive and anti-inflammatory activities [356]. In an in vitro study model, umbelliferone present in the ethanol extract of banana flower inhibited α-glucosidase, the polyol pathway, and protein glycation, and activated the peroxisome proliferator-activated receptors (PPARγ and PPARβ) thereby exhibiting anti-hyperglycemic activity [357,358]. Further, in streptozotocin-induced diabetic rats, umbelliferone at a dose of 30 mg/kg body weight showed significant glycemic control. Additionally, the antioxidant activity of umbelliferone prevented the liver cells from oxidative damage in streptozotocin-induced diabetic rats [359]. The coumarin inhibited reactive oxygen species generation, induced apoptosis, initiated cell cycle arrest and DNA fragmentation thereby exhibiting antitumor activity against liver hepatocellular cell lines [360]).

Aesculetin is reported to show radical scavenging activity in a dose-dependent manner [361]. Another study demonstrated that it exhibited the strongest scavenging activity among the ten isolated coumarins from *A. dahurica* [362]. It improved the cell viability of hydrogen peroxide-treated Caco-2 cells by enhancing mRNA expression of Nrf2 and increasing the activity of glutathione peroxidase [363].

Coumestrol exerts antioxidant activity against hydrogen peroxide-induced oxidative stress in HepG2 cells. It also prevents lipid peroxidation, ROS production and reduces SOD activity [364]. It shows an anti-inflammatory effect in LPS-activated microglia by inhibiting the production of nitric oxide, inducible nitric oxide, monocyte chemoattractant protein-1 (MCP-1), and IL-6 [365]. Yuk and co-workers studied the anti-inflammatory effects of coumestrol in lipopolysaccharide (LPS)-induced RAW264.7 macrophages and in acute lung injured mice model. Treatment with the compound reduced the production of nitric oxide (NO), tumor necrosis factor-α (TNF-α) and interleukin-6 (IL-6) in the macrophages; it further suppressed activated nuclear factor-kappa β. Furthermore, in bronchoalveolar lavage fluid of acute lung-injured mice, it reduced the level of ROS, TNF-α, MCP-1, and IL-6, inhibited NO release and suppressed the expression of NF-κB and SOD3 [366]. Moreover, its antioxidant and anti-apoptotic properties can be beneficial in treating diabetes and neurological disorders [367,368].

Bergapten, the furanocoumarin displayed moderate antioxidant activity than α-tocopherol [369,370]. In a study, the precognitive effects of bergapten attenuated the oxidative stress markers in scopolamine-induced memory impairment. It also restored the acetylcholine levels to normalcy [371]. It further provides neuroprotection in chronic constriction damage by inhibiting the overexpression of COX-2, TNF-α, and NF-ĸB [372]. In the animal model of acetic acid-induced colitis, bergapten reduced inflammation and inflammatory cell infiltration [373].

Psoralen is used in the treatment of skin diseases as it inhibits the oxidation of unsaturated lipids and prevents the impairment of barrier functions of biomembranes [374]. It even exerts strong anti-osteoporotic effects via the regulation of multiple molecular pathways such as the wnt/β-catenin, apoptosis signaling kinase 1 (ASK1)/c-jun N-terminal kinase (JNK) and the protein kinase B (AKT), and the expression of miR-488, peroxisome proliferators-activated receptor-gamma (PPARγ), and matrix metalloproteinases (MMPs) [375]. In human hepatoma cell line SMMC7721, it activates the ER signaling pathway, thereby inducing apoptosis [376]. Another study suggested that the presence of psoralen and isopsoralen in the extract of *P. corylifolia* L. induces apoptosis in four cancer cell lines (KB, KBv200, K562 and K562/ADM), contributing to its anticancer activity [377].

Eriodyctiol is a flavonoid with a wide range of biological activity. Specifically, it exhibits its antioxidant potency in various ways [378]. It modulates ROS in human keratinocyte cells [379], Nrf2, and downstream shielding phase-II enzyme activation [380], affects vanilloid receptors [379], and nitric oxide concentration, attenuates lipid peroxidation, and regulates the expression of Nrf2/HO-1, and β-glutamyl cysteine synthase pathways, among others [381]. Eriodyctiol and its prenylated derivative sigmoidin have been reported to demonstrate a comparable radical scavenging activity though the latter exhibited a higher cytotoxicity against cancer cells [382]. Through Nrf2/ARE signaling pathway activation, Eriodyctiol isolated from *Dracocephalum rupestre* PC12 cell death in a hydrogen peroxide-induced neurotoxicity [383]. By inhibiting the production of ROS, TNF-α, IL-1β, creatinine, MDA, blood urea nitrogen, TBARS among other regulating activities, Eriodyctiol has shown protective effects on cisplatin-induced kidney injury [384].

DPPH, FRAP, and ABTS antioxidant screening methods have been used to evaluate the scavenging potency of the luteolin complex, vanadium (IV) oxide sulfate monohydrate (VOSO_4_.H_2_O). The antioxidant of this flavonoid was found to increase after the formation of the complex [385]. The phospholipid complex of this flavonoid was also revealed to have a significant scavenging effect against DPPH radicals [386]. Luteolin causes apoptotic cell death in human activity by activating the HT-29 cells pathway which is mediated by mitochondria [387].

Morin protects against altered sperm parameters and testicular oxidative stress induced by bicalutamide (BCT) [388]. Morin hydrate has earlier been reported as an effective hepatic protector in both in vivo and in vitro studies [389]. It has been reported as a potential antioxidant in attenuating free radical-mediated damage to cardiovascular cells [390]. Complex formation enhances the scavenging effect of morin [391,392].

Genistein, a soya bean isoflavone, regulates the expression of antioxidant genes, modulates longevity-associated gene expression, and reduces peroxides through increased levels of MnSOD mRNA expression [393]. It has been reported to protect the kidney against IRI by attenuating both oxidative stress and inflammation [394]. As regards L-NAME-induced cardiac remodeling and dysfunction in rats, genistein has been found to be cardioprotective [395]. Genistein and daidzein have been revealed to have neuroprotective effects, enhancing choline metabolism and mitigating the PC12 cell damage induced by chlorpyrifos [396]. This study observed a better antioxidant effect for a combined therapy. Cong and colleagues reported the amelioration effect of genistein in cognitive deficits induced by chronic sleep deprivation, Nrf2 alongside downstream targets in the cortex and hippocampus of CSD-treated mice was activated. The isoflavone inhibited NF-κB, iNOS, and COX-2 activation alongside cytokines such as TNF-α, IL-6, and IL-1β [397]. Genistein improves kidney damage induced by morphine [398]. Empirical report have suggested a similar antioxidant potency for genistein and daidzein toward DNA oxidative insult [399]. As a supplement, daidzein improves embryo growth and development in early pregnancy, attributed to the isoflavone’s ability to improve the immune and antioxidant status of amniotic fluid [400]. Through the alteration of MAPK’s pathway and regulation of the inflammatory pathway in cisplatin-induced nephrotoxicity, daidzein suppresses oxidative stress and apoptosis [401]. It has also demonstrated an improved growth performance alongside antioxidant properties in weaned and growing pigs [402]. Nevertheless, daidzein has been reported to exhibit a prooxidant effect rather than being antioxidative, specifically in the brains of rats, as it decreases glutathione concentration, which weakens the body’s antioxidative defense system. It has also been suggested that the C4 keto moiety and C2 and C3 double bond in flavonoids may not be unconnected with this activity [403].

Rutin has been described as having an antioxidant activity in DPPH. It is also an inhibitor of lipid peroxidation [404]. As regards bioavailability, it has been found to elevate plasma flavonoid levels significantly [405]. In streptozotocin-induced diabetic rats, it has shown antihyperglycemic and antioxidant potency [406]. Alongside caffeic acid, it has been reported to exhibit antiaging and antioxidant potentials [407,408,409,410].

## 6. Polyphenols in Disease Management

**Figure 5:**
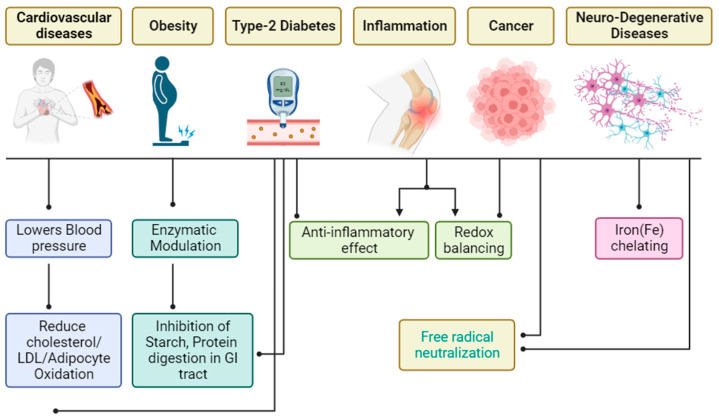
Pharmacological properties of antioxidant polyphenols in various disease models.

Polyphenols, ubiquitous in plant-derived dietary sources, exhibit profound implications in the realm of cardiovascular health, obesity, type 2 diabetes mellitus (T2DM), inflammation, cancer, and neurodegenerative diseases. Through their potent antioxidant properties, polyphenols effectively mitigate oxidative stress, thus attenuating the progression of atherosclerosis and reducing the risk of cardiovascular events. Moreover, polyphenols demonstrate promising anti-obesity effects by modulating adipocyte metabolism, promoting adipogenesis, and regulating lipid homeostasis, thereby presenting a potential therapeutic avenue in combating obesity and its associated cardiovascular complications. In the context of T2DM, polyphenols exhibit multifaceted actions, including improving insulin sensitivity, enhancing pancreatic β-cell function, and ameliorating glucose uptake, collectively contributing to glycemic control and reducing the risk of diabetic complications. Furthermore, polyphenols exert anti-inflammatory effects by suppressing pro-inflammatory cytokines and signaling pathways, thus attenuating chronic low-grade inflammation implicated in the pathogenesis of cardiovascular disease, obesity, T2DM, and various other chronic diseases, as shown in Figure 5 [411,412,413,414,415,416,417,418,419,420,421,422,423,424,425,426,427]. Additionally, polyphenols demonstrate chemo-preventive properties by inhibiting carcinogenesis, modulating cell cycle progression, inducing apoptosis, and suppressing tumor angiogenesis, highlighting their potential in cancer prevention and adjuvant therapy. Moreover, polyphenols hold promise in neuroprotection by mitigating neuroinflammation, preserving neuronal function, and inhibiting protein misfolding and aggregation, thereby potentially delaying the onset and progression of neurodegenerative diseases such as Alzheimer’s and Parkinson’s diseases [252,264]. Collectively, the multifaceted bioactivity of polyphenols underscores their therapeutic potential in mitigating the burden of chronic diseases across diverse pathological contexts [411,412,413,414,415,416,417,418,419,420,421,422,423,424,425,426,427].

### 6.1. Diabetes

Polyphenols exert their effects on diabetes through intricate molecular pathways, influencing insulin sensitivity, glucose metabolism, and related complications. Flavonoids, such as quercetin, have been implicated in enhancing insulin signaling pathways, potentially improving cellular responsiveness to insulin through the PI3K/AKT pathway [411]. Additionally, these compounds may modulate carbohydrate digestion enzymes like α-amylase and α-glucosidase, impacting postprandial glucose levels via the AMPK pathway [412].

**Figure 6:**
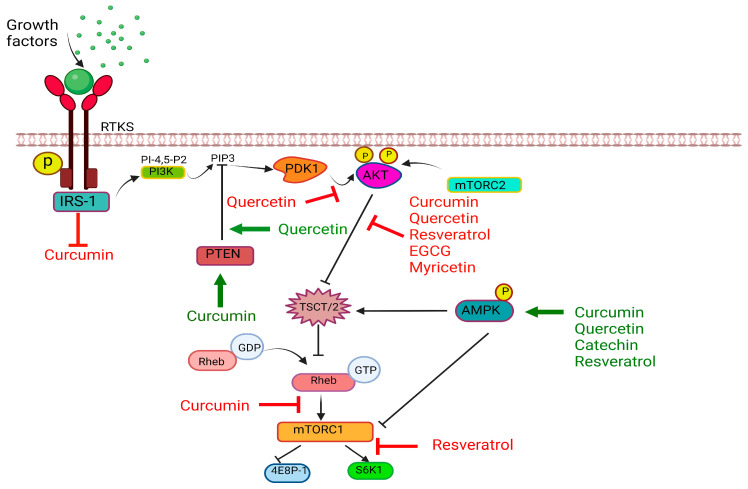
Anti-inflammatory actions of polyphenols counteracting oxidative stress in diabetes mellitus.

The anti-inflammatory actions of polyphenols involve the NF-κB pathway, potentially reducing chronic inflammation associated with insulin resistance. Their antioxidant properties, regulated by pathways like Nrf2, counteract oxidative stress, preserving pancreatic beta-cell function crucial for insulin secretion as shown in Figure 6 [413].

Polyphenols may also impact glucose homeostasis through interactions with the gut microbiota, influencing the gut-brain axis via the serotonin signaling pathway. Furthermore, in addressing diabetes complications, polyphenols could mitigate oxidative stress-related damage through the MAPK pathway, providing protective effects against diabetic nephropathy and retinopathy [413].

However, individual responses to polyphenols may vary due to factors like genetic differences and metabolism, necessitating personalized approaches. Ongoing research aims to elucidate the specific interactions between polyphenols and these pathways, offering potential insights for optimizing their integration into diabetes management strategies.

### 6.2. Respiratory Health

**Figure 7:**
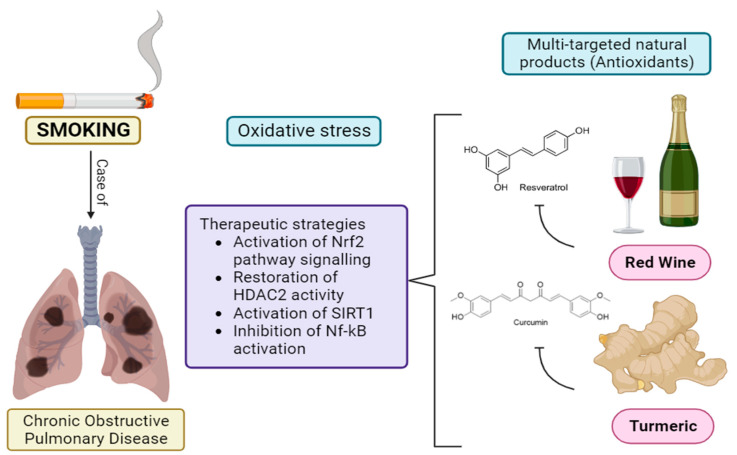
Role of polyphenols in respiratory health.

Polyphenols, prevalent in plant-derived foods, have emerged as potential contributors to respiratory health by targeting key pathways associated with inflammation and oxidative stress, as shown in Figure 7. In particular, flavonoids and phenolic acids, major polyphenols, exhibit anti-inflammatory effects by inhibiting the NF-κB pathway, suggesting a potential role in mitigating airway inflammation in conditions like asthma and COPD [414]. Additionally, their antioxidant properties, mediated through the Nrf2 pathway, could counteract oxidative stress, offering protection against respiratory damage. Certain polyphenols, such as quercetin, may influence bronchodilation by modulating calcium channels and phosphodiesterase, providing a novel approach to address airway constriction. Moreover, polyphenols may modulate immune responses and impact mucin production, suggesting potential immunomodulatory and mucolytic effects in respiratory disorders. While ongoing research seeks to elucidate the precise mechanisms, integrating polyphenol-rich foods into the diet holds promise for supporting respiratory well-being, with personalized approaches to optimize their effectiveness [415].

### 6.3. Pregnancy and Maternal Health

Polyphenolic compounds, widely distributed in a variety of plant-derived sources, have garnered significant interest due to their potential influence on maternal health and pregnancy outcomes. The intricate molecular structure of polyphenols, encompassing flavonoids, phenolic acids, and extracts rich in polyphenolic content, is currently under scrutiny for its antioxidative properties, which are crucial in alleviating oxidative stress-an established contributor to complications during pregnancy. The modulation of cellular signaling pathways, such as the nuclear factor erythroid 2-related factor 2 (Nrf2) pathway [416], is central to understanding the intricate interactions through which polyphenols exert their protective effects. Moreover, these bioactive compounds exhibit anti-inflammatory properties by inhibiting pro-inflammatory mediators and activating pathways like nuclear factor-kappa B (NF-κB), potentially influencing the inflammatory milieu during pregnancy. Additionally, polyphenols may impact vascular function by promoting endothelial health, involving the regulation of nitric oxide (NO) production and endothelial nitric oxide synthase (eNOS) activity [417]. Hormonal balance, particularly in estrogen metabolism, emerges as another facet through which polyphenols may contribute to maternal well-being. Despite encouraging findings, there remains a need for comprehensive investigations into optimal dosages, bioavailability, and specific subclasses of polyphenols pertinent to pregnancy, offering a more nuanced understanding of their therapeutic potential during this critical life stage [418].

### 6.4. Polyphenols and Microbiome-Brain Axis

In the intricate relationship known as the Microbiome-Brain Axis, polyphenols, a diverse group of bioactive compounds prevalent in various plant-based foods, have emerged as pivotal modulators. Recent research highlights the ability of polyphenols to influence microbial composition and diversity in the gut, leading to the production of bioactive metabolites, such as short-chain fatty acids (SCFAs). These metabolites, derived from microbial fermentation of polyphenols, can traverse the blood-brain barrier and impact neuronal function, potentially exerting neuroprotective effects. Polyphenols have been shown to interact with key signaling pathways, including the gut-brain axis’s intricate communication networks, involving the vagus nerve and the enteric nervous system [419]. Moreover, polyphenols demonstrate anti-inflammatory and antioxidant properties, influencing gut microbiota homeostasis and subsequently contributing to neural health. As the understanding of the Polyphenols and Microbiome-Brain Axis deepens, elucidating the specific mechanisms and pathways involved in this complex crosstalk holds significant promise for developing targeted interventions to promote brain health and potentially mitigate neurological disorders [420,421].

### 6.5. Polyphenols and Bone Health

Polyphenols, a diverse class of naturally occurring compounds found in various plant-based foods, have emerged as potential contributors to bone health, presenting a novel avenue of exploration in the field of skeletal physiology. Recent research suggests that polyphenols may influence bone metabolism through intricate mechanisms involving modulation of osteoblast and osteoclast activity. Flavonoids, a subgroup of polyphenols, have been shown to stimulate osteoblastic differentiation and mineralization, key processes in bone formation, potentially mediated through pathways such as Wnt/β-catenin signaling. Additionally, polyphenols exhibit antioxidant and anti-inflammatory properties, mitigating oxidative stress and inflammation, factors implicated in bone resorption [422]. Polyphenol-induced activation of nuclear factor erythroid 2-related factor 2 (Nrf2) pathway may also contribute to cellular defense against oxidative damage in bone cells. Furthermore, the potential impact of polyphenols on gut microbiota modulation, with implications for calcium absorption and bone mineral density, adds another layer of complexity to their role in bone health. While promising, further elucidation of the specific polyphenolic subclasses, optimal dosage, and underlying molecular pathways involved is essential for advancing our understanding and leveraging polyphenols as potential adjuncts in promoting skeletal well-being [423].

### 6.6. Polyphenols and Autoimmune Disorders

Within the realm of autoimmune disorders, the intricate interplay between polyphenols, a diverse group of bioactive compounds abundant in various plant-derived foods, has garnered increasing attention, offering a novel avenue for therapeutic exploration. Emerging research indicates that polyphenols may exert immunomodulatory effects through intricate mechanisms involving the regulation of immune cell function. Flavonoids, a prominent subclass of polyphenols, have been implicated in the suppression of pro-inflammatory cytokines, such as tumor necrosis factor-alpha (TNF-α) and interleukin-6 (IL-6), potentially mediated through the inhibition of nuclear factor-kappa B (NF-κB) signaling pathways. Moreover, polyphenols exhibit antioxidant properties, mitigating oxidative stress, a known contributor to autoimmune pathogenesis [424]. Polyphenol-induced modulation of T-helper cell balance, regulatory T-cell activity, and the suppression of autoantibody production underscore their multifaceted impact on immune regulation. The potential interplay between polyphenols and gut microbiota further adds complexity, as alterations in microbial composition may influence immune responses in autoimmune disorders. While promising, comprehensive investigations into the specific polyphenolic subclasses, dosage, and molecular pathways involved are imperative for advancing our understanding and harnessing the therapeutic potential of polyphenols in the context of autoimmune disorders [425].

### 6.7. Polyphenols and Metabolic Syndrome

Polyphenols, a diverse group of bioactive compounds ubiquitous in various plant-derived sources, have garnered considerable interest for their potential to mitigate metabolic syndrome, representing a novel avenue in the realm of metabolic health. Recent research suggests that polyphenols may exert beneficial effects through intricate pathways involved in metabolic regulation. Flavonoids, a subclass of polyphenols, have demonstrated potential in ameliorating insulin resistance, a central feature of metabolic syndrome, by modulating signaling pathways such as the phosphoinositide 3-kinase/protein kinase B (PI3K/Akt) pathway. Polyphenols also exhibit anti-inflammatory properties, targeting key mediators like nuclear factor-kappa B (NF-κB) and interleukin-6 (IL-6) [426], thereby attenuating chronic low-grade inflammation, often associated with metabolic syndrome. Furthermore, polyphenols may influence lipid metabolism, regulating pathways such as peroxisome proliferator-activated receptor gamma (PPARγ) and adenosine monophosphate-activated protein kinase (AMPK), contributing to improved lipid profiles and adipose tissue function. The potential impact of polyphenols on gut microbiota composition, affecting metabolic processes and inflammation, adds an additional layer of complexity to their role in metabolic health. While promising, comprehensive investigations into optimal dosages, specific polyphenolic subclasses, and underlying molecular mechanisms are essential for advancing our understanding and harnessing the therapeutic potential of polyphenols [427,428,429,430,431,432,433,434,435,436].

### 6.8. Polyphenols in Neurodegenerative Diseases

In the realm of neurological health, the spotlight increasingly falls on polyphenols, natural compounds abundant in a variety of dietary sources, including fruits, vegetables, and beverages like tea and wine. Due to their multifaceted properties, these bioactive molecules have emerged as promising candidates for mitigating the onset and progression of neurodegenerative diseases. With antioxidant, anti-inflammatory, and neuroprotective effects, polyphenols hold the potential to preserve neuronal function and combat the underlying mechanisms of conditions such as Alzheimer’s and Parkinson’s diseases [181,198]. Mechanistically, polyphenols exert their neuroprotective effects through various molecular pathways. They modulate oxidative stress by scavenging free radicals and upregulating antioxidant enzymes such as superoxide dismutase and catalase. Additionally, polyphenols inhibit the activation of inflammatory pathways mediated by nuclear factor-kappa B (NF-κB) and mitogen-activated protein kinases (MAPKs), thereby reducing neuroinflammation. Moreover, polyphenols interact with key proteins involved in neurodegenerative processes, such as beta-amyloid and alpha-synuclein, preventing their aggregation and fibril formation. Furthermore, polyphenols may enhance neuroplasticity and synaptic function by activating signaling pathways such as the brain-derived neurotrophic factor (BDNF) pathway. These molecular insights underscore the significance of polyphenols in the quest for effective treatments against these debilitating disorders [252,264].

## 7. Polyphenols and Epigenetics

Polyphenols, a diverse group of naturally occurring compounds in plant-derived foods, have garnered substantial attention for their potential health-promoting effects [437,438,439,440,441]. A burgeoning area of research involves the intricate interplay between polyphenols and epigenetic mechanisms, which regulate gene expression without altering the underlying DNA sequence [442,443,444,445]. The multifaceted structures of polyphenols, such as flavonoids, phenolic acids, lignans, and stilbenes, enable them to interact with enzymes involved in epigenetic modifications, such as DNA methyltransferases and histone acetyltransferases [446].

The epigenetic modulation by polyphenols encompasses several key aspects. Polyphenols may influence DNA methylation, a process crucial for gene silencing or activation. Studies have suggested that certain polyphenols possess the capacity to inhibit DNA methyltransferases, thereby affecting the methylation status of specific genes associated with various physiological processes [447].

Secondly, polyphenols can impact histone modifications, playing a pivotal role in chromatin structure and gene accessibility. Polyphenols may serve as histone deacetylase (HDAC) inhibitors, influencing the acetylation status of histones and consequently regulating gene expression. This modulation can have implications for diverse cellular functions, including cell cycle control, apoptosis, and inflammation [448].

Furthermore, polyphenols may interact with non-coding RNAs, such as microRNAs (miRNAs), which play a crucial role in post-transcriptional gene regulation. By affecting the expression levels of specific miRNAs, polyphenols can indirectly influence the expression of their target genes and, consequently, various cellular processes [449].

The impact of polyphenols on epigenetic processes holds promise for therapeutic applications. Understanding the epigenetic mechanisms influenced by polyphenols may offer insights into their potential roles in preventing or managing diseases with an underlying epigenetic component, such as cancer, cardiovascular diseases, and neurodegenerative disorders [450].

Despite the promising avenues of research, it is important to acknowledge the complexity of these interactions. The bioavailability, metabolism, and specific molecular targets of polyphenols can vary, influencing their efficacy in epigenetic modulation. Thus, further elucidation of the specific polyphenolic compounds, dosage, and duration required for optimal epigenetic effects is imperative for harnessing their full therapeutic potential. Exploring the dynamic interplay between polyphenols and epigenetics remains a captivating frontier in nutritional and medical sciences, holding promise for personalized interventions and developing novel strategies for health promotion and disease prevention [451].

## 8. Polyphenols, Food and Commercial Importance

### 8.1. Polyphenols in Food Processing

The effects of food processing techniques on polyphenols, vital bioactive compounds in plant-derived foods, are pivotal considerations in shaping the content, structure, and bioavailability of these compounds and, consequently, their potential health benefits. Heat treatment, including cooking and pasteurization, can lead to the thermal breakdown of certain polyphenols, but moderate heat can enhance extractability. Extraction methods, such as solvent extraction or supercritical fluid extraction, profoundly influence the types and concentrations of polyphenols in the final product [428]. Fermentation processes generate both metabolites with potential health benefits and may cause the breakdown of specific polyphenolic structures. Storage conditions, exposing foods to light, air, and elevated temperatures, can contribute to polyphenol degradation. Moreover, these processing techniques influence the bioavailability of polyphenols, affecting their absorption, distribution, metabolism, and excretion within the human body [429].

While certain methods may result in polyphenol loss, others enhance bioavailability or generate bioactive metabolites. A comprehensive understanding of the intricate interactions between food processing and polyphenols is crucial for optimizing the health benefits of polyphenol-rich foods and informing dietary recommendations for promoting processed foods that retain or enhance their polyphenolic content [430].

### 8.2. Utilizing Polyphenols from Food Waste

**Figure 8:**
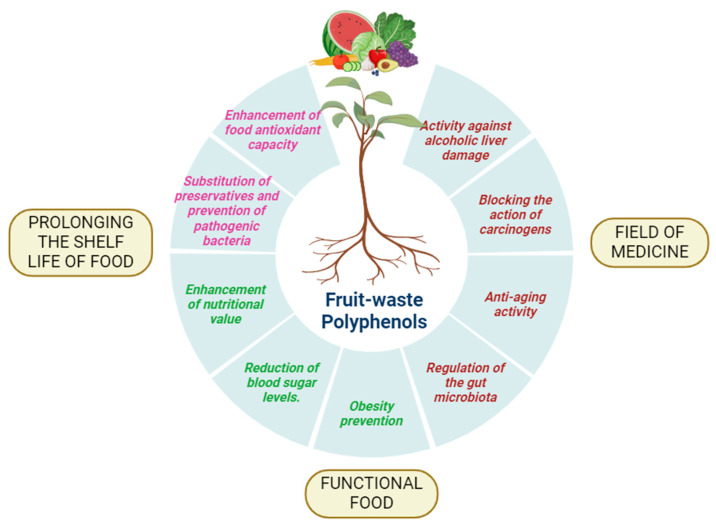
Extracting polyphenols from food waste represents an innovative approach.

Deriving polyphenols from waste food stands as a pioneering strategy, capitalizing on resource efficiency and environmental conservation as shown in Figure 8. Food waste, replete with bioactive compounds, emerges as a reservoir rich in potentially valuable polyphenols [431,432,433]. Examples of extraction avenues from diverse food waste streams include:

- Fruit and vegetable peelings: The outer layers of commonly discarded fruits and vegetables house a substantial concentration of polyphenols. Peelings, especially those sourced from apples, citrus fruits, carrots, and potatoes, can be extracted through diverse extraction methodologies [434,435];
- Wine residues: Post-wine production, pomace comprising grape skins, seeds, and stems retains polyphenols. Extracting these compounds from wine residue diminishes waste and yields polyphenolic extracts with applications across various industries [436,437];
- Coffee grounds: Residual polyphenols persist in used coffee grounds after coffee brewing. Extracting polyphenols from spent coffee grounds presents a sustainable avenue for waste utilization [438,439];
- Tea residues: Spent tea leaves or residue post-brewing represent an additional source of polyphenols. Employing suitable extraction techniques enables the recovery of polyphenols from these discarded tea remnants [440,441];
- Waste from fruit and vegetable processing: By-products generated during the processing of fruits and vegetables, encompassing peels, seeds, and cores, often become discarded waste. These by-products can be investigated as potential sources of polyphenols through adept extraction processes [442];
- Brewing and distillation of by-products: Residues ensuing from brewing beer or distilling spirits, such as spent grains, may contain polyphenols. Employing innovative extraction methods facilitates the recovery of polyphenols from these by-products [443,444];

This polyphenol extraction paradigm from food waste is congruent with the principles of a circular economy, emphasizing waste minimization and maximal resource utilization. It is imperative to ensure that extraction methods align with sustainability principles and that resultant polyphenolic extracts adhere to stringent safety and quality standards for prospective applications in industries such as food, pharmaceuticals, or cosmetics. Concurrently, focused endeavors should be directed toward the development of efficient and eco-friendly extraction processes to scale this approach feasibly.

## 9. Conclusions

Consumption of plant-based products, depending on their phyto-antioxidant content, reduces the risk of development and progression of many chronic diseases and improves human health. In conclusion, our comprehensive review has elucidated the substantial health advantages associated with the consumption of plant-based products enriched in phyto-antioxidants. This dietary modality has demonstrated a discernible reduction in the susceptibility to and progression of diverse chronic diseases, thereby substantiating its potential as a cornerstone in augmenting human health.

Although the predominant paradigm attributes the salutary effects to the antioxidant activity inherent in polyphenols, it is imperative to acknowledge the intricate and incompletely understood mechanisms of action, particularly concerning specific phenolic compounds. Mitigating this lacuna in knowledge necessitates a nuanced exploration of polyphenol bioavailability, with the anticipation that such insights will conduce to a more sophisticated comprehension of their health-propagating attributes.

Expanding the purview of our investigation, we have probed the multifaceted roles of polyphenols in disease management. Specifically, we have scrutinized their potential impact on conditions such as diabetes, respiratory health, pregnancy, maternal health, autoimmune disorders, and metabolic syndrome. This nuanced examination of polyphenols in diverse pathophysiological contexts enhances our grasp of their versatile applications and underscores their potential as therapeutic modalities in varied health paradigms.

Furthermore, our inquiry extends to the realm of polyphenols in food processing, wherein we have scrutinized their influence on the nutritional profile and bioactivity of processed foodstuffs. A foray into the burgeoning field of polyphenols in epigenetics has augmented our understanding of their putative influence on gene expression and cellular homeostasis.

Recognizing the imperatives of sustainability, we have addressed the deployment of polyphenols derived from residual food sources. This facet not only mitigates food wastage but also unravels novel avenues for harnessing the health benefits of polyphenols from unconventional reservoirs.

Notwithstanding the strides made in polyphenol research, challenges persist, notably the impact of structural variations on optimal bioavailability, metabolic kinetics, and modes of biological activity. Accordingly, ongoing investigations are dedicated to structural modifications of polyphenols, with the overarching aim of enhancing their bioavailability and therapeutic efficacy. This strategic pursuit portends to unlock the full therapeutic potential of polyphenols, offering precision interventions for disease prevention and management, thereby cementing the pivotal role of plant-based products in fortifying holistic human well-being. The comprehensive exploration of these diverse facets underscores the exigency for sustained research and accentuates the potential of polyphenols as invaluable contributors to human health across a spectrum of conditions.

The hypothesis underlying the importance of the comprehensive review posits that a thorough exploration and synthesis of existing literature on polyphenols in plant-based products enriched with phyto-antioxidants is pivotal for advancing our understanding of their profound impact on human health. This hypothesis is rooted in the premise that by meticulously examining the diverse roles and mechanisms of action of polyphenols across various chronic diseases and physiological processes, the review serves as a cornerstone for elucidating the intricate interplay between dietary factors and health outcomes. Furthermore, the hypothesis suggests that by critically analyzing the current state of knowledge, identifying gaps, and proposing future research directions, the comprehensive review not only contributes to scientific knowledge but also informs clinical practice, dietary guidelines, and public health initiatives aimed at promoting optimal health and preventing chronic diseases. Ultimately, the hypothesis underscores the indispensable role of comprehensive reviews in synthesizing evidence, fostering scientific discourse, and driving advancements in research and healthcare practices.

## Figures and Tables

**Table 1:** Classification of polyphenols.

Sr. No.	Polyphenolic Class	Category	Example	References
1.	Phenolic acid	Hydroxybenzoic acid	Gallic acid	[37]
		Hydroxycinnamic acids	*p*-coumaric acid	[38]
			Caffeic acid	[39]
			Ferulic acid	[40]
			Sinapic acid	[41]
2.	Flavanoids	Isoflavones, Neoflavones and Chalcones	Genistein and daidzein	[42]
		Flavones, Flavonols, Flavanones and Flavanonols	Quercetin and Kaempferol	[43]
		Flavanols and Proanthocyanidins	Catechins	[44]
		Anthocyanins		[45]
3.	Stilbenes		Resveratrol	[28,46]
4.	Tanins	Condensed tannins		[30,31]
		Hydrolysable tannins		
		Complex tannins		
		Phlorotannins		
5.	Lignans		Matairesinol	[47]
			Secoisolariciresinol	[48]
6.	Coumarins		Umbelliferone	[36]
			Aesculin	
			Psoralen	

**Table 3:** Pharmacological activity of polyphenols with in vitro/in vivo models.

Polyphenols	In Vitro/In Vivo Model	Pharmacological Activity	References
Red Wine Extracts: Malvidin-3-glucoside Petunidin-3-glucoside Malvidin-3-cumaroylglucoside	HT-29 cells	Red wine extract inhibits cytokine-induced IkB degradation. Red wine extract inhibits COX-2 induction by cytokines. Red wine extract inhibits iNOS induction by cytokines.	[147]
Pomegranate Juice Extract: Punicalagin Ellagic acid	Liposome model HT-29 cells	Inhibition of cell proliferation of nonmetastatic colon cancer cells. Induced apoptosis in HT-29 cells.	[148]
Apple Polyphenol Extracts: Catechins Chlorogenic acid	MKN 28 cells Male Wistar rats	Prevention of xanthine-xanthine oxidase-induced cell injury. Helps inhibit ROS-induced lipid peroxidation. Significantly prevents indomethacin injury in vivo as well as in vitro.	[149]
Phenolic Acids: Chlorogenic acid 4-coumaroylquinic acid Flavonols: Quercetin-3-rutinoside Quercetin-3-rhamnoside	HT-29 cells CaCo-2 cells	Exhibit preventive effectiveness in colon cell lines.	[150]
Procyanidins: Procyanidin dimer Procyanidin trimer C1	IPEC-1 cells TOPIG hybrid pigs	Resulted in a decrease in Lipid peroxidation. It helps increase the total antioxidant status.	[151]
Anthocyanins: Delphinidin 3-galactoside Cyanidin 3-galactoside	CaCo-2 cells	Exhibit an intracellular antioxidant activity, thereby protecting cells from ROS.	[152]
Green Tea Polyphenols: Epigallocatechin gallate	BALB/c mice with DSS-induced colitis	Inhibit the activity of nuclear factor-κB. Help lower the concentration of TNF-α.	[153]
Tomato Extract: Resveratrol Quercetin	C57/BL6 mice with DSS-induced colitis	May be involved in the inhibition of inflammatory factors. May help promote the production of proteins utilized in tissue repair.	[154]

## Data Availability

All data is included in the article.
